# Developing the Healthy and Competitive Organization in the Sports Environment: Focused on the Relationships between Organizational Justice, Empowerment and Job Performance

**DOI:** 10.3390/ijerph18179142

**Published:** 2021-08-30

**Authors:** Suk-Kyu Kim, Yunduk Jeong

**Affiliations:** 1 Department of Sports Science, Dongguk University, Gyeongju 38066, Korea; skkim2018@dongguk.ac.kr; 2 College of General Education, Kookmin University, Seoul 02707, Korea

**Keywords:** organizational justice, empowerment, job performance, professional sports teams, healthy organization

## Abstract

As improving the job performance of employees is becoming increasingly significant for organizational growth, a major challenge for organizational development managers is to understand and explore the important antecedents of job performance. Therefore, the purpose of this study was to examine the structural relationships between organizational justice, empowerment, and job performance in the South Korean professional sports industry. Recently, many professional sports teams in South Korea have attempted to improve employees’ job performance for the future survival of the teams. The research participants were 371 employees affiliated with 40 male professional sports teams. The validity and reliability of the measures involved were investigated by carrying out confirmatory factor, Cronbach’s alpha, and correlation analyses. A structural equation-modeling test with a maximum likelihood estimation was performed to evaluate the structural relationships between distributive justice, procedural justice, interactional justice, empowerment and job performance, and the mediating effects of empowerment. The findings revealed the positive impacts of (a) distributive justice on empowerment, (b) procedural justice on empowerment, (c) interactional justice on empowerment, (d) procedural justice on job performance, and (e) interactional justice on job performance. Furthermore, empowerment fully mediated the relationship between interactional justice and job performance. These findings highlight the importance of increasing organizational justice and empowering employees when managing professional sports organizations.

## 1. Introduction

In South Korea, professional sports were introduced to distract people’s attention from politics and provide healthy recreational activities to the public [1]. At that time, South Korean corporate giants promoted professional sports teams as a component of the government’s pressure and marketing for public relations. Professional baseball and soccer were founded in 1982 and 1983, respectively. Professional baseball, which began with the slogan “Dreams and hopes for children”, became the most popular sport in Korea. Since the establishment of pro basketball and volleyball in 1997 and 2004, respectively, spectator sports have attracted attention from diverse groups of people, creating a sports culture that leads to popular culture [2]. With the emergence of new sports stars in a short period, the public has become enthused with professional sports because of their stellar performance [3]. As of 2021, four leagues—baseball, soccer, basketball, and volleyball—are still active in Korea. 

Most U.S. and European professional sports teams are in the form of profit-making corporations. In contrast, South Korean professional sports teams depend heavily on financial support from their parent companies. The parent companies provide approximately 70–80% of the budgets. Recently, however, many professional sports teams are under financial difficulties because their parent corporations have reduced their financial support for the professional sports teams. According to Jeong, Kim, Kim, and Zhang [4], there is a major reason for the reduced financial support. Although the parent companies provide considerable funding to professional sports teams every year, they perceive that the use of professional sports teams as promotional tools is ineffective compared to the past. Today, the parent companies prefer marketing communications via various social media, such as YouTube, Facebook, and Instagram, or social contribution activities to utilizing professional sports teams. To cope with the challenge, making profits is becoming an essential task for the future survival of professional sports teams [4]. Thus, it is essential to improve the employees’ job performance and explore the factors that influence job performance and contribute to the future success of professional sports teams. 

Many researchers have long been interested in the importance of justice in the personal satisfaction of employees and the efficient functioning of the organization [5,6,7]. This is because the justice in which members are engaged in various organizational tasks is coupled with their motivation, job performance, empowerment, and organizational citizenship behavior [8]. Justice theory has been studied by scholars from Adams’ [9] distributive justice study that the organizational compensation affects job satisfaction and performance. Thibaut and Walker [10] developed several important frameworks connecting procedural justice in the context of legal disputes, and systematic research was conducted by presenting differentiated conceptions from distributive justice. Bies and Moag [11] also attempted to differentiated from procedural justice by introducing the concept of interactional justice. In sum, organizational justice is divided into three parts: distributive, procedural, and interactional justice. 

In addition, according to Jeong [12], improving individual empowerment could lead to job performance because the autonomous participation and commitment of employees are related to their job activities. In this regard, many scholars have focused on the concept of empowerment [13]. In community psychology, human resources, and organizational behavior, empowerment has been viewed as the interaction of the individual and the organization, which means that empowerment is likely to be affected by organizational justice [14]. Additionally, Deci and Ryan [15] claimed that one could improve the organization’s performance, dedication, and contribution by managing the self-determination power of the members depending on what culture and degree of justice the company emphasizes, which means that job performance is likely to be affected by empowerment. Thus, we hypothesized the effect of organizational justice on empowerment and empowerment on job performance in this study.

The contribution of this study is twofold. First, the present study examined the mediating effects of empowerment on the relationship between organizational justice and job performance. Previous studies on organizational justice tended to prioritize the direct effects of organizational justice and job performance [16]. On the other hand, based on previous studies, in examining the relationship between organizational justice and job performance, empowerment is likely to be operationalized as a key intervening construct. Next, in organizational justice literature, although a large amount of literature is devoted to a general corporation [17,18,19,20] (the most common type of corporate structure), little research has been conducted on sports corporations, particularly professional sports teams. The current study focuses on sports corporations, such as professional sports teams. 

Accordingly, with the aid of recent academic explorations of professional sports, this study examined the structural relationships between organizational justice, empowerment, and job performance. Moreover, the present study explored the mediating effects of empowerment on the relationship between organizational justice and job performance, which could broaden the understanding of sports organizations. 

## 2. Literature Review, Research Hypotheses, and Model

### 2.1. Organizaional Justice

Organizational justice research has been carried out on organizational decision-making according to organizational behavior and effective in accordance with the development of society, climate, and equity [21]. These streams of research on organizational justice are considered important factors in the study of ethical aspects, such as an ethical climate. This is the study of equity that began with Aristotle’s Nicomachean ethics in ancient Greece and is related to the normative approach as a semantic point of morality and righteousness [22]. Equity, from this classical perspective, refers to equal distribution. Equity has been studied from the perspectives of justice and distribution, but the focus of such research has shifted from an individual perspective to an organizational perspective [23]. Adams [9] presented organizational justice (distributive justice) theory, which other researchers have developed continuously. In addition, organizational justice research has been developed in a wide range of studies by the study that justice perception is an important factor in individual satisfaction and organizational efficiency [24]. In this respect, based on James [25], organizational justice is defined broadly as “the individual’s and the group’s perception of the fairness of treatment received from an organization and their behavioral reaction to such perception” (p. 269, [26]). While organizational justice can be defined clearly, however, the contents of fairness itself are multidimensional, and thus, various perspectives and approaches are being studied. Early work on organizational justice emphasized the meaning of distribution, and procedural justice studies were then conducted to measure the fair distribution of such compensation. By expanding this compensation and distribution, the relationship between the members was transformed, and the study of organizational justice from the perspective of the interaction has expanded.

In this regard, organizational justice is generally divided into three types: distributive justice, procedural justice, and interactional justice. Distributive justice is defined as the fairness of outcomes, such as wages, compensation, and promotion. Individuals tend to consider distributive justice by comparing their outcome/input ratio with that of their colleagues [9,27]. Homans [28], an early distributive justice researcher, argued that individuals who participate in exchange relationships recognize distributive justice when they are compensated for the expenses they have invested. The importance of procedural justice was overlooked in the early days of equity theory. This is because equity theory emphasizes distributive justice by focusing on what is fair in terms of improving the job satisfaction of members. On the other hand, empirical studies of procedural justice have shown that fair procedures can mitigate the negative effects of inadequate compensation [29]. Leventhal [30] reported that if the distribution process seems fair, it could be accepted as fair, even if there is a disadvantage in the final determined distribution. Therefore, the study of Greenberg and Folger [31] clarified the significance of applying the concept of procedural justice to the organizational situation in earnest. Research on interactional justice began with the criticism that by the mid-1980s, organizational justice research had been limited to distributive and procedural justice while neglecting the aspect of interactions. Bieg and Moag [11] first suggested the concept of interactional justice by indicating that existing studies on procedural justice neglected the context or situation, in which fairness problems occur in the organization. In other words, the treatment received from others should be considered as an important factor in the perception of fairness. In particular, Bieg and Moag [11] discussed the importance of considering truthfulness, respect, propriety, and justification in judging interactional justice. Hence, interactional justice should be considered an important variable among organizational justice subfactors [32,33]. 

### 2.2. Empowerment

The concept of empowerment is used widely in various disciplines, such as sociology, politics, business administration, social welfare, education, and psychology. Empowerment does not have a clear definition in the literature because it is used in various sociocultural environments and political contexts. Zimmerman [34] stated that insisting on a single definition of empowerment is like giving a formula or prescription. This is contrary to the concept of empowerment itself, which is inherently multifaceted. On the other hand, terms that frequently appear in the discourse on empowerment include self-strength, control, mastery, self-power, self-reliance, choice, decision making, freedom, independence, awakening, and capability [35]. These concepts are all closely related to empowerment. Tengland (p. 90, [36]) defined the form of a conceptual discussion about empowerment: “A change (internal or external to the person) is an increase in empowerment if it is an increase in a person’s control over the determinants of their quality of life, through (necessarily) an increase in either health (e.g., through self-confidence, self-esteem, self-efficacy, and autonomy), or knowledge (self-knowledge, raising consciousness, skills development, and competence), or freedom (negative or positive).” In addition, Page and Czuba [37] took a multidimensional approach to understand the concept of empowerment. Empowerment is multidimensional because it can exist at the social, psychological, political, and economic levels and various other levels, including the individual, organizational, community, and national levels [38].

Thomas and Velthouse [39] described empowerment as increased intrinsic task motivation and suggested four cognitions of empowerment: sense of impact, competence, meaningfulness, and choice. Based on Thomas and Velthouse [39], Spreitzer [40] suggested four components of empowerment: meaning, competence, self-determination, and impact. Meaning is the value of a business goal or purpose that is determined in relation to an individual’s ideas or standards [39]. In other words, it is a concept that includes a consensus among the values, beliefs, and behaviors required for a task and makes the individual feel that the job given in the organization is worth the effort. Thus, an empowered individual feels that their work is important and interesting [41]. Competence is the individual’s belief in their ability to perform a skillful activity [42]. The social cognitive theory of Bandura considers this an important dimension. A higher job performance competence means more positive job satisfaction and job performance [43]. Self-determination refers to an individual’s belief that they can control themselves without interference from others [44]. Self-determined individuals can take action that is more decisive because they believe that their behavior is caused by their own decisions [45]. This represents a strong sense of ownership of the task and personal responsibility for and commitment to the outcome. The impact is the extent to which individuals believe that they can influence the strategic, administrative, or operating outcomes at work [46]. Thus, in an organizational context, self-determination refers to the degree of control individual employees feel they have over their jobs. In contrast, impact refers to their perception of control over organizational outcomes. 

### 2.3. Research Hypotheses Development

According to previous studies, empowerment increases when employees perceive fairness within an organization. For example, Kuokkanen et al. [47] examined the relationship between organizational justice and empowerment among 2152 nurses in Finland and reported that organizational justice and empowerment had a clear correlation. Choo and Bae [48] examined the correlation between organizational culture, organizational justice, empowerment, and organizational effectiveness among public officials in South Korea. They demonstrated that distributive justice, procedural justice, and interactional justice positively correlated with empowerment. Hong [49] empirically analyzed how the organizational fairness perceived by revenue officers affects empowerment and employee efforts. They confirmed that a high level of organizational fairness resulted in a high level of empowerment. Kirkman, Shapiro, Novelli, and Brett [50] indicated that a self-managed team, a group of people responsible and accountable for managing the team, planning and scheduling the workflow, production-related decisions, and problem solving [51], depended on a multidimensional justice perspective. Thus, the perceptions of organizational justice are closely related to the attitudes and behaviors of the organizational members [50].

**Hypothesis** **1-1** **(H1-1):** *Distributive justice will positively influence empowerment.*

**Hypothesis** **1-2** **(H1-2):** *Procedural justice will positively influence empowerment.*

**Hypothesis** **1-3** **(H1-3):** *Interactional justice will positively influence empowerment.*

Many studies reported that organizational justice is likely to influence job performance. For example, Krishnan, Loon, Binti, Ahmad, and Yunus [52] explored the role of organizational justice on the job performance of employees. They indicated a positive association between distributive, procedural, and interactional justice on the employees’ job performance. Shan, Ishaq, and Shaheen [53] examined the impact of organizational justice on job performance and reported that all three kinds of organizational justice predict job performance. Nasurdin and Khuan [54] examined the influence of organizational justice (distributive and procedural justice) on job performance (task and contextual performance), and regression analysis showed that distributive justice was related to task performance. Moreover, procedural justice had a significant impact on contextual performance. Therefore, organizational justice affects job performance. Accordingly, this study tested the following hypotheses:

**Hypothesis** **2-1** **(H2-1):** *Distributive justice will positively influence job performance.*

**Hypothesis** **2-2** **(H2-2):** *Procedural justice will positively influence job performance.*

**Hypothesis** **2-3** **(H2-3):** *Interactional justice will positively influence job performance.*

Mixed evidence exists suggesting a relationship between empowerment and job performance. Chiang and Hsieh [55] explored the impacts of perceived organizational support and empowerment on job performance and reported that empowerment positively affected job performance. Tetik [56] examined the effects of empowerment on job satisfaction and job performance and found that empowerment led to improved job performance. Hechanova, Alampay, and Franco [57] also argued that empowerment was positively correlated with job performance. Based on the empirical perspective described in the literature, this study tested the following hypothesis:

**Hypothesis** **3** **(H3):** *Empowerment will positively influence job performance.*

With respect to the mediating effect of empowerment on the relationship between organizational justice and job performance, previous studies suggested that organizational justice is likely to influence both empowerment and job performance [47,48,49,50,51,52,53,54]. In addition, based on existing studies, empowerment is likely to affect job performance [55,56,57]. Therefore, based on former studies, the present study asserts that empowerment could mediate the relationship between organizational justice and job performance. Hence, the following hypotheses were tested.

**Hypothesis** **4-1** **(H4-1):** *Empowerment will mediate the relationship between distributive justice and job performance.*

**Hypothesis** **4-2** **(H4-2):** *Empowerment will mediate the relationship between procedural justice and job performance.*

**Hypothesis** **4-3** **(H4-3):** *Empowerment will mediate the relationship between interactional justice and job performance.*

**Figure 1:**
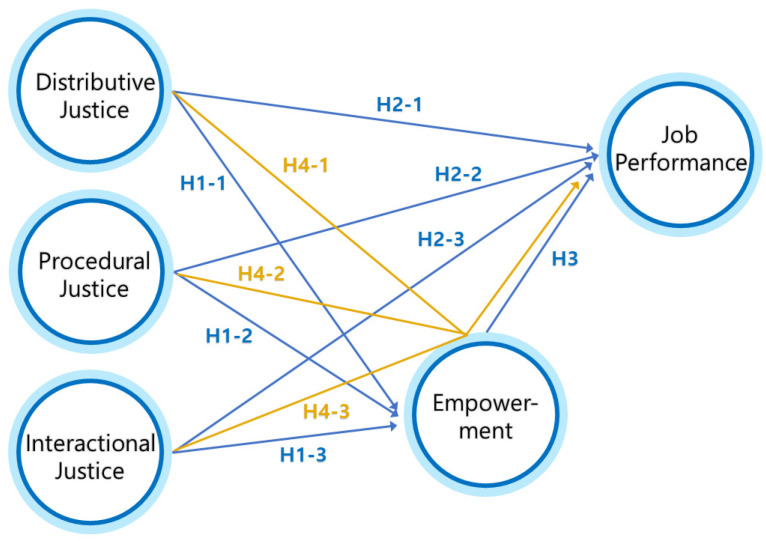
Proposed conceptual model.

Based on a thorough review of previous studies, the current study proposes the following conceptual model (Figure 1).

## 3. Method

### 3.1. Data Collection

The purpose of this study was to examine the structural relationships between organizational justice, empowerment, and job performance. A sample should represent the entire population. Thus, it is important to confirm the characteristics of a good sample. A panel of three sports, organizational behavior, and human resource management professors suggested that a sample design should be goal-oriented, proportional, and economical. Based on these standards, the present study tried to collect a sample that adequately reflects the population. Generally, structural equation modeling requires a minimum of 200 respondents for effective parameter estimation [58]. The research hypothesis was verified by collecting data using a purposive sampling technique from front office employees at 40 male professional sports teams in South Korea, including 17 soccer teams, 10 baseball teams, 8 basketball teams, and 5 volleyball teams from 1 September to 15 November 2020. These teams reportedly focused on improving organizational justice. The surveys were not conducted through a visiting survey because of the spread of COVID-19. Instead, the surveys were administered by email or Kakao Talk (the most widely used messaging app for smartphones and personal computers in South Korea) procedures using Google surveys. The author contacted the front office managers, and the questionnaires were distributed to the employees’ email or Kakao Talk ID upon agreement. In the case of surveys, the purpose of the questionnaire was explained to the participants, and the questionnaire was answered using a self-administration method. Four hundred respondents completed a self-administered questionnaire, but 29 questionnaires were incomplete and were eliminated. The remaining 371 responses were analyzed. The demographic characteristics included basic personal information, such as gender (69.3% male and 30.7% female); age (27.8% were in their 20s, 45% were in their 30s, and 27.2% were in their 40s or older); administrative department (34.5% public relations and marketing, 31.8% management support, 12.4% team (athletes) operation and support, and 21.3% other departments); and level of education (6.2% high school, 9.4% associated degree, 75.2% university, and 9.2% graduate). 

### 3.2. Measures and Data Analyses

The theoretical relationships among organizational justice, empowerment, and job performance were assessed. The survey instrument was modified and adapted using the scales from existing studies. The questionnaire consisted of four main sections: (a) organizational justice, (b) empowerment, (c) job performance, and (d) demographic information. Organizational justice is structured as follows. In the case of distributive justice, Moorman [59], Niehoff and Moorman [60], and Díaz-Gracia, Barbaranelli, and Jiménez [61] were referenced. Procedural justice was also based on the research by Moorman [59], Niehoff and Moorman [60], and Díaz-Gracia et al. [61]. Interactional justice was based on the research of Moorman [59], and Niehoff and Moorman [60]. Each of these three measures consisted of four items. The organizational justice scale developed by Niehoff and Moorman [55] was adopted and modified in this study because the scale was a reliable and valid instrument, according to Gürbüz and Mert [62]. 

In the case of empowerment, Spreitzer [40] and Hochwälder and Brucefors [63] were referenced. The meaning, competence, self-determination, and impact comprised four items each, giving a total of 16 items. According to Hochwälder and Brucefors [63], the empowerment scale has been assessed with respect to its main psychometric properties in only two studies, Spreitzer [40] and Kraimer Seibert and Liden [64]. The current study adopted the scale to measure empowerment because the psychometric properties of Spreitzer’s empowerment scale in the review of sports organization studies [4,12] was considered satisfactory. Finally, four items from Jeong [12] were used to measure job performance. 

All items were measured using a five-point Likert scale, ranging from 1 (strongly disagree) to 5 (strongly agree). A panel of three sports organizational behavior and human resource management professors was invited to clarify these items and ensure content validity. Based on their feedback, the preliminary questionnaire was modified and improved for final adoption and distribution. Data collected using the questionnaire were analyzed using the SPSS 24.0 and AMOS 24.0 software packages. SPSS 24.0 was used for frequency analysis, reliability analysis, correlation analysis. AMOS 24.0 was used for confirmatory factor analysis and structural equation modeling. 

### 3.3. Validity and Reliability

The present study used confirmatory factor analysis (CFA) to confirm the dimensionality of the measurement model using the maximum likelihood estimation via AMOS version 24.0. The goodness-of-fit indices of CFA showed a satisfactory fit with the data (NFI = 0.902, RFI = 0.963, IFI = 0.936, TLI = 0.900, CFI = 0.935, and RMSEA = 0.061) (Table 1), all of which were within the recommended thresholds [65]. The convergent validity was confirmed by calculating the factor loadings, construct reliability (CR), and average variance extracted (AVE) based on the measurement model. As listed in Table 1, all factor loading values (0.506–0.887) except for the item ‘procedural justice 4′ were statistically significant (*p* < 0.001) and greater than the cutoff value of 0.5 [66]. The value of the item ‘procedural justice 4′ was 0.300 and was removed. All CR values (0.777–0.904) exceeded the minimum requirement of 0.7, and all AVE values (0.509–0.759) were above the minimum of 0.5 [67] (Table 1). The results showed strong evidence of convergent validity. 

According to the Fornell and Larcker criterion [67], discriminant validity has been established: (1) the AVE of the latent variable was greater than the square of the correlation between the latent variables, and (2) each item loads highest on its associated construct. The discriminant validity in this study was investigated by comparing the square root of AVE for each construct with the correlations between the pairs of latent variables [62]. For discriminant validity, the diagonal elements in Table 2 should be greater than that of the off-diagonal elements [67]. Comparing all correlation coefficients with the square roots of AVEs in Table 2, the results indicated satisfactory discriminant validity. In addition, Table 2 shows confidence intervals for correlations. The reliability estimates (Cronbach’s alpha) for distributive justice, procedural justice, interactional justice, meaning, competence, self-determination, impact, and job performance (0.705–0.870) were above the recommended threshold of 0.7, suggesting that the measures were reliable [67] (Table 1). 

## 4. Results

### 4.1. Model Fit and Structural Model

Structural equation modeling (SEM) was examined to test the hypothesized relationships. All goodness-of-fit indices of the structural model indicated that the model achieved an acceptable fit (NFI = 0.902, IFI = 0.936, TLI = 0.900, CFI = 0.935, and RMSEA = 0.063) [68]. Hypotheses 1, 2, and 3 were tested. As shown in Table 3, the relationships between the distributive justice and empowerment (0.227, *p* < 0.05), procedural justice and empowerment (0.214, *p* < 0.05), and interactional justice and empowerment (0.435, *p* < 0.001) were established, supporting hypotheses 1-1, 1-2 and 1-3. A nonsignificant path emerged for distributive justice → job performance; thus, hypothesis 2-1 was rejected. On the other hand, the paths from procedural justice to empowerment and from interactional justice to job performance were positive and statistically significant (0.216 and 0.203, *p* < 0.05 and *p* < 0.01), thereby supporting hypotheses 2-2 and 2-3. Empowerment had a significant positive effect on job performance (0.320, *p* < 0.001), supporting hypothesis 3.

### 4.2. Mediating Effect of Empowerment

This study followed Baron and Kenny’s [69] general guidelines and tested the significance of indirect effects using Preacher and Hayes’s [70] bootstrap procedure to examine the mediating effects of empowerment. The bootstrap sample is the same as the original dataset. The number of repetitions (number of bootstrap samples) was 500 times. 

As shown in Table 4, in hypothesis 4-1 (distributive justice → empowerment → job performance), the relationship coefficient decreased from 0.156 to 0.123 when empowerment was incorporated into the model as a mediator. On the other hand, the direct effect without a mediator was not significant, and the indirect effect of distributive justice on the job performance via empowerment was not significant, while the confidence interval included zero (CI = −0.007 to 0.228), rejecting hypothesis 4-1. 

In hypothesis 4-2 (procedural justice → empowerment → job performance), the relationship coefficient decreased from 0.301 to 0.216 when empowerment was incorporated into the model as a mediator. Nevertheless, the indirect effect of procedural justice on job performance via empowerment was not significant while the confidence interval included zero (CI = −0.002 to 0.219); hence, hypothesis 4-2 was rejected (see Table 4). 

In hypothesis 4-3 (interactional justice → empowerment → job performance), the relationship coefficient decreased from 0.388 to 0.203. The direct effect was not significant when empowerment was incorporated into the model as a mediator. Moreover, the indirect effect of interactional justice on job performance via empowerment was significant while the confidence interval did not include (CI = 0.028 to 0.328), supporting hypothesis 4-3 and indicating full mediation (see Table 4). 

## 5. Discussion 

### 5.1. Theoretical Implications

Based on the results, the current study provided several theoretical implications.

This study contributes to general or sports organizational behavior studies by uncovering the mediating effect of empowerment on the relationship between interactional justice and job performance. Existing studies in various fields failed to consider the mediating effects of empowerment on the relationship between organizational justice and job performance [16]. This study examined the indirect effects of three dimensions of organizational justice on job performance via empowerment. The mediating effects of empowerment were not found because the indirect effects between distributive justice and job performance and between procedural justice and job performance were not significant. On the other hand, the present study found that empowerment fully mediated the relationship between interactional justice and job performance, indicating that employees must be empowered to improve job performance. This result helps bridge the gap in the literature by exploring the detailed effects of interactional justice on job performance through empowerment. Hence, based on our findings, managers who pursue interactional justice should not overlook the importance of empowerment in building employees’ job performance. 

While realizing the relationship between organizational justice and empowerment, this study showed that organizational justice plays a crucial role in improving empowerment in sports organizational behavior. Hence, empowerment increases when employees perceive a fair outcome distribution (e.g., pay and feedback), fairness of the decision-making processes, and decision-making treatment and communication within an organization. With respect to pay, if an organization introduces and pursues a fair wage differential policy, i.e., a difference in wages between employees according to their skills, employees are likely to work hard in the expectation of a higher salary. In other words, employees are likely to be always highly involved in organizational work. This viewpoint aligns with previous studies. For example, Lee, Kim, and Kim [71] examined the influence of perceived organizational justice on empowerment, organizational commitment, and turnover intention and indicated that organizational justice led to empowerment. Similarly, other studies support this argument [47,48,49,50]. Therefore, to improve employees’ empowerment, it is important to maintain fairness within the organization, particularly ‘a wage differential’.

The present study confirmed that interactional justice is the most influential factor in predicting empowerment. The relationship between organizational justice and empowerment has been controversial in the context of organizational behavior during the past several decades. For example, Lee [72] examined the relationships between organizational justice, leader–member exchange, empowerment, and service-oriented organizational citizenship behavior. They reported that ‘distributive justice’ was the most influential factor in improving empowerment. In contrast, Ham [73] tested the effect of perceived organizational justice on empowerment. They showed that ‘procedural justice’ was the most influential factor in increasing empowerment, which is in line with Lee [72]’ study. On the other hand, Hong [49] empirically analyzed how organizational fairness affected empowerment and employee efforts and showed that ‘interactional justice’ was the most influential factor in building empowerment. Previous studies show a very different outcome for the relationship. This study confirmed that interactional justice was the most potent factor in predicting empowerment. Thus, strengthening interactional justice should be a priority for increasing empowerment. 

Distributive justice did not affect job performance, whereas procedural justice and interactional justice significantly impacted job performance. These findings mean that job performance increases when employees can voice their opinion about the task process and when employees are provided with explanations for decisions and are being treated with dignity and respect by employers [74]. These findings are aligned with previous studies suggesting that organizational justice affects job performance. For example, Al Rawashdeh [75] investigated organizational justice and its impact on job performance and reported that providing employees with incorporeal support and establishing the organizational climate will improve the employees’ performance. Ali, Abdullah, Othayman, and Ali [76] explored the mediation role of leader–member exchange between organizational justice and job performance. They proved empirically that procedural and interactional justice were positive and significantly correlated with job performance. Therefore, procedural justice and interactional justice are indicators of job performance. 

This study heeds existing researchers’ claim that empowerment helps improve job performance. More specifically, Yilmaz [77] examined the probable effect of perceived empowerment on job performance in the tourism sector as front-line employees and reported that all four dimensions of empowerment affected employee job performance. Arslan, Zaman, and Phil [78] explored the effect of empowerment on job performance in the software sector, revealing empowerment had a significant positive impact on the job performance of project professionals. In the context of sport management, Kim, Won, and Kwag [79] investigated the relationships between leader–member exchange quality, empowerment, and job performance of fitness center instructors, and they reported that employee empowerment had a positive effect on job performance. In other words, when employees possess high levels of empowerment, they could have a strong sense of responsibility for the task process, which enables them to work hard. Therefore, sport managers should focus on improving employees’ empowerment to promote employees’ job performance. 

### 5.2. Practical Implications

The key findings of this study have important organizational behavior or human resource management implications for sports organizations.

First, fair pay for staff is particularly important [80,81]. In considering fairness in wages, the concept of ‘differentiation of salary’ is important. ‘Fairness of salary’ and ‘differentiation of salary’ are significantly different. While ‘fairness of salary’ can be argued to reduce employees’ interest in salary and focus on creating performance instead, ‘differentiation of salary’ is used to encourage employees to pay attention to salary and work for a higher salary [82,83]. Because the purposes of ‘fairness of salary’ and ‘differentiation of salary’ are different, the methods to implement them also must differ. In the case of ‘differentiation of salary’, it is advantageous to decide the salary at the enterprise level because the comparison target should be made as much as possible, and the difference should be felt. On the other hand, in the case of ‘fairness of salary’, it is important not to compare members with each other but rather to effectively communicate the message of fairness to the individual within the frame set by the company. Thus, it becomes important for the manager to direct opinion on the salary decision rather than the rating grade.

Second, boosting effective organizational communication between employers and employees is important for fostering organizational justice. When employers use effective communication, employees can have increased trust in the organization and perceive organizational justice. For example, suppose employers or managers explain in minute detail the standards for incentive and promotion system for employees in accordance with their performance. Employees can accept these standards, which can result in perceptions of organizational justice. 

Third, another predictor of organizational justice is employee participation. When actively encouraged to participate in decision-making processes concerning organizational procedures, employees can increase their perceptions of justice, even when the outcome is not in the employees’ favor [84]. According to Kernan and Hanges [85], when employees are given a voice in the organizational decision-making process, they perceive procedural justice and interactional justice. Thus, to develop organizational justice, team managers should always heed the employees’ voices. 

Many professional sports teams in South Korea embraced the hierarchy culture in the past [4]. They were almost similar to bureaucratic organizations, such as the military. Such hierarchical nature of Asian cultures has prevented employees’ innovation or creativity from being accepted in these organizations. Therefore, it is necessary to improve the empowerment of professional sports team members to increase job performance. To develop employees’ empowerment, managers should inspire employees with sufficient autonomy in organizational work [4]. Managers tend to micromanage or oppose new ideas of employees in every way because they believe that employees have insufficient work experience. As a result, employees feel a surge of enthusiasm for their work which is ineffective and inefficient. Thus, managers should dispose of authoritarianism and provide employees with decision-making opportunities. In addition, managers should encourage employees through appropriate praise. Praise, along with communication and empathy, increases employees’ morale and enthusiasm, which can lead to solid individual growth [86]. David Novak, the former CEO of YUM! Brands Inc., stated that “compliments are the most powerful weapon that an organization’s leaders can use, and most people are thirsty for recognition, and creating a work environment where all employees are valued is a direct path to success” [4]. 

## 6. Conclusions

As we have seen, the main purpose of this study was to examine the relationships between distributive justice, procedural justice, interactional justice, empowerment, and job performance with emphasis on the mediating effect of empowerment on the relation between organizational justice and job performance in the context of sports organizational behavior. Findings showed significant impacts of distributive justice on empowerment; procedural justice on empowerment; interactional justice on empowerment; procedural justice on job performance; interactional justice on job performance; empowerment on job performance; and demonstrated that empowerment fully mediated relationship between interactional justice and job performance. The current study added to the growing body of research that isolated the mediating effect of organizational justice on job performance via empowerment. It is hoped that managers should develop organizational justice and encourage employee’s empowerment to improve their job performance.

Despite the findings of the present study, there were some limitations. First, the proposed model included a limited number of constructs. Hence, in future research, to broaden the understanding of sports employee behavior, it will be meaningful to include various exogenous variables (e.g., organizational culture, support, or leadership) influencing empowerment and job performance [4] and a key moderating variable. Second, because the current study was limited to male professional sports teams, further attention should be paid to incorporating the research results into female professional sports teams. Recently, due to the rising popularity of the women’s volleyball league in South Korea and throughout the world, many researchers are paying attention to the league’s development. Lastly, the results of the present study did not support the relationships between distributive justice and job performance, and the mediating effects between ‘distributive justice and job performance’, and ‘procedural justice and job performance’ via empowerment. Because the characteristics of this study’s samples are different from those of the samples in prior organizational behavior studies, future research should investigate the relationships using other characteristics of the study samples. 

## Figures and Tables

**Table 1:** Summarized results for the validity and reliability assessments.

Scale Items	Standardized Loadings	CR	AVE	Cronbach’s α
**Distributive justice**				
Employees receive fair compensation according to their efforts and abilities.	0.579	0.801	0.509	0.720
Employees receive fair compensation as well as work performance.	0.508			
Employees receive fair compensation as equivalently as assigned responsibilities.	0.730			
Employees are rewarded for their degree of work experience.	0.760			
**Procedural justice**				
The organization has a procedure to provide feedback on the results of the reward.	0.851	0.904	0.759	0.870
The organization has a procedure for accurate individual compensation.	0.833			
The organization tends to listen to employees’ views regarding rewards.	0.813			
**Interactional justice**				
The boss tends to respect my opinion in respect of decisions as to rewards.	0.778	0.811	0.523	0.740
In making decisions regarding compensation, the boss tries to rule out personal prejudices.	0.544			
The boss is thoughtful and attentive to my decision concerning compensation.	0.578			
In making decisions regarding compensation, the boss treats me in a straightforward manner.	0.648			
**Meaning**				
My job performance is personally meaningful.	0.887	0.881	0.714	0.851
I am confident that I shall accomplish my business objectives.	0.832			
I have autonomy in deciding how to do business.	0.716			
**Competence**				
I feel rewarded for what I do.	0.828	0.824	0.612	0.800
I want to achieve higher goals than others.	0.784			
I can control what happens in my department.	0.654			
**Self-determination**				
What I do is important within the organization.	0.698	0.777	0.537	0.705
I can accomplish my work goals.	0.658			
I maintain my opinion while doing business.	0.644			
**Impact**				
My job is important to improve my career.	0.848	0.827	0.620	0.791
I am confident that I will push ahead with what I plan to in my organization.	0.815			
I can make important decisions about the way organization works.	0.592			
**Job performance**				
I am actively engaged in performing my duties.	0.856	0.811	0.527	0.779
I think my job performance contributes to the development of the team.	0.655			
I tend to get recognition from my boss for performing my duties.	0.765			
My job performance level is high compared to my colleagues.	0.506			

NFI = 0.902, RFI = 0.963, IFI = 0.936, TLI = 0.900, CFI = 0.935, and RMSEA = 0.061.

**Table 2:** Correlations between the constructs.

	**DJ**	**PJ**	**IJ**	**Empowerment**	**JP**
**DJ**	**0.849**				
**PJ**	0.614 **	**0.933**			
**IJ**	0.260 **	0.230 **	**0.860**		
**Empowerment**	0.514 **	0.470 **	0.424 **	**0.978**	
**JP**	0.504 **	0.454 **	0.424 **	0.578 **	**0.883**
			**95.0% Confidence Interval**		
**Parameter**		**Estimate**	**Lower**	**Upper**	**P**
**DJ ←→ PJ**		0.614	0.533	0.695	0.000
**DJ ←→ IJ**		0.260	0.161	0.359	0.000
**DJ ←→ Empowerment**		0.514	0.426	0.602	0.000
**DJ ←→ JP**		0.504	0.416	0.593	0.000
**PJ ←→ IJ**		0.230	0.130	0.329	0.000
**PJ ←→ Empowerment**		0.470	0.379	0.560	0.000
**PJ ←→ JP**		0.454	0.363	0.545	0.000
**IJ ←→ Empowerment**		0.424	0.331	0.516	0.000
**IJ ←→ JP**		0.424	0.332	0.517	0.000
**Empowerment ←→ JP**		0.578	0.495	0.662	0.000

** *p* < 0.01, DJ: Distributive justice, PJ: Procedural justice, IJ: Interactional justice, and JP: Job performance.

**Table 3:** Structural parameter estimates.

Hypothesis	Path	Standardized Coefficient	C.R.	Supported?
1-1	Distributive justice → Empowerment	0.227	2.454 *	Yes
1-2	Procedural justice → Empowerment	0.214	2.514 *	Yes
1-3	Interactional justice → Empowerment	0.435	7.215 ***	Yes
2-1	Distributive justice → Job performance	0.123	1.259	No
2-2	Procedural justice → Job performance	0.216	2.395 *	Yes
2-3	Interactional justice → Job performance	0.203	2.941 **	Yes
3	Empowerment → Job performance	0.320	4.403 ***	Yes

* *p* < 0.05, ** *p* < 0.01, *** *p* < 0.001.

**Table 4:** Mediation analysis.

Path.	Direct Effects without Mediator	Direct Effects with a Mediator (CI)	Indirect Effects (CI)	*p*	Mediation Hypotheses
Distributive justice → empowerment → job performance	0.156	0.123 (−0.110 to 0.796)	0.073 (−0.007 to 0.228)	0.067	Not supported
Procedural justice → empowerment → job performance	0.301 ***	0.216 (−0.181 to 0.457)	0.069 (−0.002 to 0.219)	0.060	Not supported
Interactional justice → empowerment → job performance	0.388 ***	0.203 (−0.057 to 0.459)	0.139 * (0.028 to 0.328)	0.016	Supported (Full mediation)

* *p* < 0.05, *** *p* < 0.001; bootstrap confidence in parentheses, CI = confidence interval.

## References

[1] Jeong Y., Kim S.K., Yu J.G. (2021). Examining the Process behind the Decision of Sports Fans to Attend Sports Matches at Stadiums Amid the SARS-CoV-2 Pandemic: The Case of South Korea. Sustainability.

[2] Jeong Y. (2019). The role of motivation, involvement, and experience in forming team loyalty of professional soccer supporters. Korean J. Sport Manag..

[3] Lee J.Y., Ko J.H. (2012). Spectator’s value cognition and expected-benefit factors on professional baseball sport star. Korean J. Phy. Edu..

[4] Jeong Y., Kim E., Kim M., Zhang J.J. (2019). Exploring relationships among organizational culture, empowerment, and organizational citizenship behavior in the South Korean professional sport industry. Sustainability.

[5] Cohen-Charash Y., Spector P.E. (2001). The role of justice in organizations: A meta-analysis. Organ. Behav. Hum. Decis. Process..

[6] Isaac J.E. (2001). Performance related pay: The importance of fairness. J. Ind. Relat..

[7] Lee C., Law K.S., Bobko P. (1999). The importance of justice perceptions on pay effectiveness: A two-year study of a skill-based pay plan. J. Manag..

[8] Greenberg J. (1990). Organizational Justice: Yesterday, Today, and Tomorrow. J. Manag..

[9] Adams J.S. (1965). Inequity in social exchange. Advances in Experimental Social Psychology.

[10] Thibaut J.W., Walker L. (1975). Procedural Justice: A Psychological Analysis.

[11] Bies R., Moag R., Lewicki R.J., Sheppard B.H., Bazerman M.H. (1986). Interactional justice: Communication criteria of fairness. Research on Negotiation in Organizations.

[12] Jeong Y.D. (2017). Structural Relationship among Organizational Culture, Empowerment, and Job Performance, and Comparison of Models-Focused on Professional Football Corporate Club and Citizen Club. Ph.D. Thesis.

[13] Bose I., Emirates U.A. (2018). Employee empowerment and employee performance: An empirical study on selected banks in UAE. J. Appl. Manag. Investig..

[14] Aujoulat I., d’Hoore W., Deccache A. (2007). Patient empowerment in theory and practice: Polysemy or cacophony?. Patient Educ. Couns..

[15] Deci E.L., Ryan R.M. (2010). Self-Determination.

[16] Jameel A.S., Ahmad A.R., Mousa T.S. (2020). Organizational justice and job performance of academic staff at public universities in Iraq. Sky. Bus. J..

[17] Moazzezi M., Sattari S., Bablan A.Z. (2014). Relationship between organizational justice and job performance of Payamenoor University Employees in Ardabil Province. Singap. J. Bus. Econ. Manag. Stud..

[18] Akram T., Lei S., Haider M.J., Hussain S.T. (2020). The impact of organizational justice on employee innovative work behavior: Mediating role of knowledge sharing. J. Innov. Knowl..

[19] Novitasari D., Asbari M., Wijaya M.R., Yuwono T. (2020). Effect of Organizational Justice on Organizational Commitment: Mediating Role of Intrinsic and Extrinsic Satisfaction. Int. J. Sci. Manag. Stud. (IJSMS).

[20] Jung H.J., Ali M. (2017). Corporate social responsibility, organizational justice and positive employee attitudes: In the context of Korean employment relations. Sustainability.

[21] Ambrose M.L., Schminke M. (2009). The role of overall justice judgments in organizational justice research: A test of mediation. J. Appl. Psychol..

[22] Ward A. (2010). Justice as economics in Aristotle’s Nicomachean ethics. Can. Political Sci. Rev..

[23] Mayer D.M., Bardes M., Piccolo R.F. (2008). Do servant-leaders help satisfy follower needs? An organizational justice perspective. Eur. J. Work. Organ. Psychol..

[24] Greenberg J. (1990). Employee theft as a reaction to underpayment inequity: The hidden cost of pay cuts. J. Appl. Psychol..

[25] James K. (1993). The social context of organizational justice: Cultural, intergroup, and structural effects on justice behaviors and perceptions. Justice in the Workplace: Approaching Fairness in Human Resource Management.

[26] Aryee S., Budhwar P.S., Chen Z.X. (2002). Trust as a mediator of the relationship between organizational justice and work outcomes: Test of a social exchange model. J. Organ. Behav..

[27] Colquitt J.A., Scott B.A., Judge T.A., Shaw J.C. (2006). Justice and personality: Using integrative theories to derive moderators of justice effects. Organ. Behav. Hum. Decis. Process..

[28] Homans G. (1961). Social Behavior: Its Elemental Forms.

[29] Greenberg J., Tyler T.R. (1987). Why procedural justice in organizations?. Soc. Justice Res..

[30] Leventhal G.S. (1980). What should be done with equity theory?. Social Exchange.

[31] Greenberg J., Folger R. (1983). Procedural justice, participation, and the fair process effect in groups and organizations. Basic Group Processes.

[32] He W., Fehr R., Yam K.C., Long L.R., Hao P. (2017). Interactional justice, leader–member exchange, and employee performance: Examining the moderating role of justice differentiation. J. Organ. Behav..

[33] Fouquereau E., Morin A.J., Huyghebaert T., Chevalier S., Coillot H., Gillet N. (2020). On the value of considering specific facets of interactional justice perceptions. Front. Psychol..

[34] Zimmerman M.A. (1990). Taking aim on empowerment research: On the distinction between individual and psychological conceptions. Am. J. Community Psychol..

[35] Oladipo S.E. (2009). Psychological empowerment and development. Edo J. Couns..

[36] Tengland P.A. (2008). Empowerment: A conceptual discussion. Health Care Anal..

[37] Page N., Czuba C.E. (1999). Empowerment: What is it. J. Ext..

[38] Peterson N.A. (2014). Empowerment theory: Clarifying the nature of higher-order multidimensional constructs. Am. J. Community Psychol..

[39] Thomas K.W., Velthouse B.A. (1990). Cognitive elements of empowerment: An “interpretive” model of intrinsic task motivation. Acad. Manag. Rev..

[40] Spreitzer G.M. (1995). Psychological empowerment in the workplace: Dimensions, measurement, and validation. Acad. Manag. J..

[41] Quinn R.E., Spreitzer G.M. (1997). The road to empowerment: Seven questions every leader should consider. Organ. Dyn..

[42] Gist M.E. (1987). Self-efficacy: Implications for organizational behavior and human resource management. Acad. Manag. Rev..

[43] Spreitzer G.M., Kizilos M.A., Nason S.W. (1997). A dimensional analysis of the relationship between psychological empowerment and effectiveness satisfaction, and strain. J. Manag..

[44] Deci E.L., Connell J.P., Ryan R.M. (1989). Self-determination in a work organization. J. Appl. Psychol..

[45] Bono J.E., Judge T.A. (2003). Self-concordance at work: Toward understanding the motivational effects of transformational leaders. Acad. Manag. J..

[46] Ashforth B.E. (1989). The experience of powerlessness in organizations. Organ. Behav. Hum. Decis. Process..

[47] Kuokkanen L., Leino-Kilpi H., Katajisto J., Heponiemi T., Sinervo T., Elovainio M. (2014). Does organizational justice predict empowerment? Nurses assess their work environment. J. Nurs. Scholarsh..

[48] Choo J.Y., Bae J.H. (2016). An analysis of the structural relationship of organizational culture, organizational justice, empowerment and organizational effectiveness using structural equation models. Crisisonomy.

[49] Hong S.B. (2009). Influence of organizational fairness perceived by revenue officers on empowerment and employee efforts. J. Korea Contents Assoc..

[50] Kirkman B.L., Shapiro D.L., Novelli L., Brett J.M. (1996). Employee concerns regarding self-managing work teams: A multidimensional justice perspective. Soc. Justice Res..

[51] Wellins R.S., Wilson R., Katz A.J., Laughlin P., Day C.R., Price D. (1990). Self-Directed Teams: A Study of Current Practice.

[52] Krishnan R., Loon K.W., Yunus N.A.S. (2018). Examining the relationship between organizational justice and job performance. Int. J. Acad. Res. Bus. Soc. Sci..

[53] Shan S., Ishaq H.M., Shaheen M.A. (2015). Impact of organizational justice on job performance in libraries. Libr. Manag..

[54] Nasurdin A.M., Khuan S.L. (2007). Organizational justice as an antecedent of job performance. Gadjah Mada Int. J. Bus..

[55] Chiang C.F., Hsieh T.S. (2012). The impacts of perceived organizational support and psychological empowerment on job performance: The mediating effects of organizational citizenship behavior. Int. J. Hosp. Manag..

[56] Tetik N. (2016). The effects of psychological empowerment on job satisfaction and job performance of tourist guides. Int. J. Acad. Res. Bus. Soc. Sci..

[57] Hechanova M.R.M., Alampay R.B.A., Franco E.P. (2006). Psychological empowerment, job satisfaction and performance among Filipino service workers. Asian J. Soc. Psychol..

[58] Hair J.F., Black W.C., Babin B.J., Anderson R.E., Tatham R.L. (2005). Multivariate analysis of data. Porto Alegre Bookm..

[59] Moorman R.H. (1991). Relationship between organizational justice and organizational citizenship behaviors: Do fairness perceptions influence employee citizenship?. J. Appl. Psychol..

[60] Niehoff B.P., Moorman R.H. (1993). Justice as a mediator of the relationship between methods of monitoring and organizational citizenship behavior. Acad. Manag. J..

[61] Díaz-Gracia L., Barbaranelli C., Jiménez B.M. (2014). Spanish version of Colquitt’s organizational justice scale. Psicothema.

[62] Gürbüz S., Mert I.S. (2009). Validity and reliability testing of organizational justice scale: An empirical study in a public organization. Rev. Public Adm..

[63] Hochwälder J., Brucefors A.B. (2005). A psychometric assessment of a Swedish translation of Spreitzer’s empowerment scale. Scand. J. Psychol..

[64] Kraimer M.L., Seibert S.E., Liden R.C. (1999). Psychological empowerment as a multidimensional construct: A test of construct validity. Educ. Psychol. Meas..

[65] Hooper D., Coughlan J., Mullen M.R. (2008). Structural equation modelling: Guidelines for determining model fit. Electron J. Bus. Res. Methods.

[66] Woo J.P. (2014). The Concept and Understanding of Structural Equation Model.

[67] Fornell C., Larcker D.F. (1981). Evaluating structural equation models with unobservable variables and measurement error. J. Mark. Res..

[68] Tabachnick B.G., Fidell L.S. (2007). Experimental Designs Using ANOVA.

[69] Baron R.M., Kenny D.A. (1986). The moderator–mediator variable distinction in social psychological research: Conceptual, strategic, and statistical considerations. J. Personal. Soc. Psychol..

[70] Preacher K.J., Hayes A.F. (2008). Asymptotic and resampling strategies for assessing and comparing indirect effects in multiple mediator models. Behav. Res. Methods.

[71] Lee K.E., Kim J.H., Kim M.J. (2016). Influence of perceived organizational justice on empowerment, organizational commitment and turnover intention in the hospital nurses. Indian J. Sci. Technol..

[72] Lee Y.S. (2014). Effect of domestic airline companies’ organizational justice on the quality of LMX, empowerment and the service oriented organizational citizenship behavior. Foodserv. Ind. J..

[73] Ham K.H. (2017). Effects of Perceived Organizational Justice on Empowerment, Organizational Commitment and Turnover Intention of Food Material Distribution Employee. Master’s Thesis.

[74] Colquitt J.A., Scott B.A., Rodell J.B., Long D.M., Zapata C.P., Conlon D.E., Wesson M.J. (2013). Justice at the millennium, a decade later: A meta-analytic test of social exchange and affect-based perspectives. J. Appl. Psychol..

[75] Al Rawashdeh E.T. (2013). Organizational justice and its impact upon job performance in the Jordanian customs department. Int. Manag. Rev..

[76] Ali Z., Abdullah N.H., Othayman M.B., Ali M. (2019). The role of LMX in explaining relationships between organizational justice and job performance. J. Comput..

[77] Yilmaz O.D. (2015). Revisiting the impact of perceived empowerment on job performance: Results from front-line employees. Turizam.

[78] Arslan M., Zaman R., Phil M. (2014). Effect of empowerment on job performance: A study of software sector of Pakistan. Res. Humanit. Soc. Sci..

[79] Kim T.J., Won D.Y., Kwag M.S. (2016). The relationships between leader-member exchange quality, empowerment, and job performance of the fitness center instructors. Sports Sci. Res..

[80] Charles A. (2011). Fairness and wages in Mexico’s maquiladora industry: An empirical analysis of labor demand and the gender wage gap. Rev. Soc. Econ..

[81] Temnitskii A.L. (2007). Fairness in wages and salaries as a value orientation and a factor of motivation to work. Sociol. Res..

[82] Birnbaum M.H. (1983). Perceived equity of salary policies. J. Appl. Psychol..

[83] Rees A. (1993). The role of fairness in wage determination. J. Labor Econ..

[84] Bies R.J., Shapiro D.L. (1988). Voice and justification: Their influence on procedural fairness judgments. Acad. Manag. J..

[85] Kernan M.C., Hanges P.J. (2002). Survivor reactions to reorganization: Antecedents and consequences of procedural, interpersonal, and informational justice. J. Appl. Psychol..

[86] Seon Y.Y., Jeong Y.D. (2020). Analysis of the core competencies of Taekwondo instructors: Using Delphi Technique. J. Korean Sports Assoc..

